# Breath Hydrogen Gas Concentration Linked to Intestinal Gas Distribution and Malabsorption in Patients with Small-bowel Pseudo-obstruction

**DOI:** 10.4137/bmi.s2139

**Published:** 2009-01-15

**Authors:** Yoshihisa Urita, Toshiyasu Watanabe, Tadashi Maeda, Yosuke Sasaki, Susumu Ishihara, Kazuo Hike, Masaki Sanaka, Hitoshi Nakajima, Motonobu Sugimoto

**Affiliations:** 1 Department of General Medicine and Emergency Care, Toho University School of Medicine, Omori Hospital, Tokyo, Japan; 2 Department of Hematology, Toho University School of Medicine, Omori Hospital, Tokyo, Japan

**Keywords:** breath hydrogen gas, intestinal gas volume, plain abdominal radiograph, malabsorption

## Abstract

**Background:**

The patient with colonic obstruction may frequently have bacterial overgrowth and increased breath hydrogen (H2) levels because the bacterium can contact with food residues for longer time. We experienced two cases with intestinal obstruction whose breath H2 concentrations were measured continuously.

**Case 1:**

A 70-year-old woman with small bowel obstruction was treated with a gastric tube. When small bowel gas decreased and colonic gas was demonstrated on the plain abdominal radiograph, the breath H2 concentration increased to 6 ppm and reduced again shortly.

**Case 2:**

A 41-year-old man with functional small bowel obstruction after surgical treatment was treated with intravenous administration of erythromycin. Although the plain abdominal radiograph demonstrated a decrease of small-bowel gas, the breath H2 gas kept the low level. After a clear-liquid meal was supplied, fasting breath H2 concentration increased rapidly to 22 ppm and gradually decreased to 9 ppm despite the fact that the intestinal gas was unchanged on X-ray. A rapid increase of breath H2 concentration may reflect the movement of small bowel contents to the colon in patients with small-bowel pseudo-obstruction or malabsorption following diet progression.

**Conclusions:**

Change in breath H2 concentration had a close association with distribution and movement of intestinal gas.

## Background

Most of the intestinal gas is produced by anaerobic fermentation of unabsorbed carbohydrates in the right colon.^1^ When unabsorbed carbohydrate reaches the caecum, it is rapidly fermented to short chain acids by anaerobic colonic bacteria, liberating carbon dioxide (CO_2_), hydrogen (H2), and, in some people, methane (CH4), which diffuse rapidly into the blood and is exhaled into the breath. Because bacteria represent the sole source of gut H2 and CH4, fasting breath H2 and CH4 gases have been used as markers of colonic fermentation.^2^ As H2 production increases when a small amount of carbohydrate is supplied to colonic bacteria, the measurement of breath H2 concentration has been proposed as an indicator of carbohydrate malabsorption.^3^ The patient with colonic obstruction or delayed small intestinal transit may frequently have bacterial overgrowth and increased breath H2 levels because the bacterium can contact with food residues for longer time. We experienced two cases with intestinal obstruction whose breath H2 concentrations were measured continuously. Change in breath H2 concentration had a close association with distribution and movement of intestinal gas. We hereby report a close association between breath H2 concentration and intestinal gas imaging detected on plain abdomen radiographs in patients with intestinal organic or functional obstruction.

## Method of Measuring Hydrogen Concentration

Breath H2 levels were measured using Breath Gas Analyzing Device model TGA-2000 (TERAMECS CoLtd, Kyoto, Japan). TGA 2000 H2 gas analyzing device is the same theory of gas chromatography analysis method.^4^ Indoor (ambient) air is used for carrier gas. In order to make ideal carrier gas, the indoor air goes through desiccating agent tubes two times before contacting sensor. First of all, the indoor air goes through desiccating agent tube by pumping. While the pump is working, gas flow regulation valve is opened. Carrier gas keeps going forward to the gas sensor with gas X-ray procedure discharging outlet. Necessary volume of the breath gas having H2 is manually sucked by hypodermic syringe from the collection bag. Then the breath gas is manually injected into six-way-valve unit including sample-loop section for the breath-gas holding. With manually push sampling valve, the breath gas at sample-loop section carrier gas will go forward to a molecular sieve via separation column.

Before breath gas analyzing test, inject standard-gas taken by hypodermic syringe into TGA-2000. The standard-gas is to use for calibration of correct volume of gas analyzing. After calibration, breath gas analyzing process is started. It will take 3 minutes to complete the gas measurement of each sample. Response of H2 gas concentration from the gas sensor is calculated by both measuring circuit and a computer system, and the results are displayed on the monitor.

## Case Report

### Case 1

**Figure 1:**
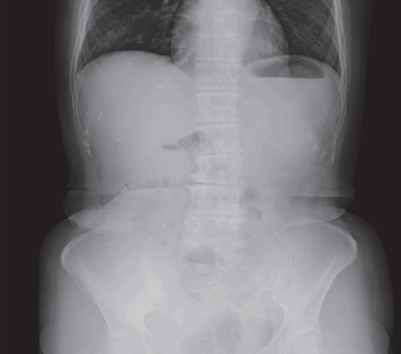
Abdominal plain radiograph of case 1 on admission showed a markedly dilated small bowel, with no dilatation of the colon.

**Figure 2:**
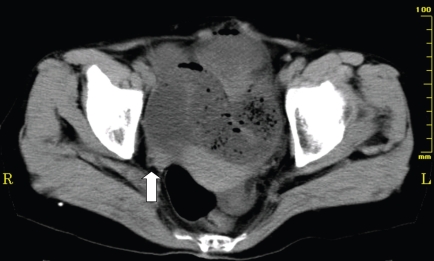
An abdominal computerized tomography (CT) showed largely distended small bowel loops and a smooth transition zone (arrow) at the site of obstruction.

A 70-year-old woman presented with abdominal pain and distention. She has a past history of surgical treatment for peritonitis before 13 years. Plain abdominal radiograph showed a markedly dilated small bowel, with no dilatation of the colon (Fig. 1). An abdominal computerized tomography (CT) showed largely distended small bowel loops and a smooth transition zone (arrow) at the site of obstruction (Fig. 2). Fasting breath H2 concentration was 3 ppm at that time.

**Figure 3:**
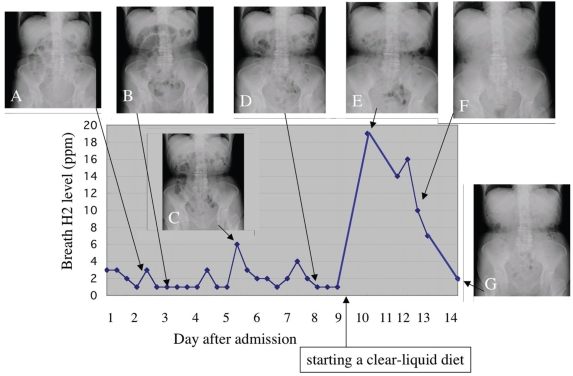
Clinical course of case 1. Changes in breath hydrogen concentration and abdominal plain radiographs on representative dates are demonstrated.

The patient was treated with the use of a gastric tube for drainage of gastric juice. Nutrition was provided intravenously. During the first four days, although small bowel gas increased gradually (Figs. 3A, 3B), breath H2 concentrations remain less than 4 ppm (Fig. 3). On the fifth day after admission, small bowel gas decreased and a small amount of colonic gas was demonstrated on the plain abdominal radiograph (Fig. 3C). The breath H2 concentration at that time increased from 1 to 6 ppm and reduced again to the baseline the next day. This value kept the low level (1–2 ppm) from the hospital days 5 through 8. Since intestinal gas was gradually reduced on radiographic imaging (Fig. 3D), a liquid meal was supplied at noon on the ninth hospital day. The breath H2 concentration on the next day increased markedly to 19 ppm despite she had no abdominal symptoms and intestinal gas was not increased on the plain abdominal radiograph (Fig. 3E). Although diets progressed from liquids to solids as tolerated, breath H2 concentrations were decreased to 2 ppm 5 days after supplying meals. The patient did not complain of any abdominal symptoms during a routine progressive diet and intestinal gas decreased continuously (Figs. 3F, 3G).

### Case 2

**Figure 4:**
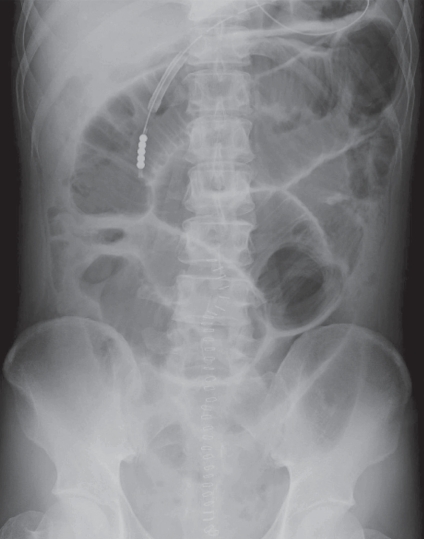
Abdominal plain radiograph of case 2 on admission showed multiple dilated gas-filled small-bowel loops, with no dilatation of the colon one day after undergoing surgical treatment.

The patient is a 41-year-old man who received surgical treatment for appendicitis. One day after undergoing surgical treatment, the patient developed diarrhea and abdominal distention. X-ray demonstrated multiple dilated gas-filled small-bowel loops, with no dilatation of the colon (Fig. 4). In spite of increased intestinal gas, breath H2 concentration held between 1 and 3 ppm.

**Figure 5:**
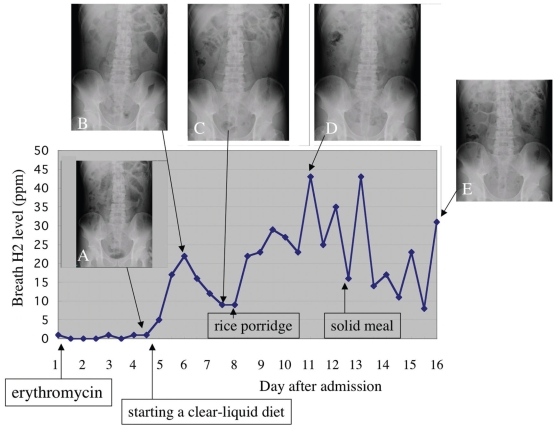
Clinical course of case 2 after a treatment with erythromycin. Changes in breath hydrogen concentration and abdominal plain radiographs on representative dates are demonstrated.

Since intestinal gas was not reduced after treatment with the use of an ileus tube for two weeks, the patient was treated with intravenous administration of 250 mg of erythromycin twice a day. Three days after the beginning of the treatment (Fig. 5A), the plain abdominal radiograph demonstrated a decrease of small-bowel gas and a clear-liquid meal was served after removal of an ileus tube. Next morning fasting breath H2 concentration increased rapidly to 22 ppm and gradually decreased to 9 ppm after 5 days. During those five days, the intestinal gas was unchanged (Figs. 5B, 5C). Then, full-liquid diets were offered in small amounts to make sure the patient can tolerate them. Two days after diet progression to rice porridge, fasting breath H2 concentration increased to 44 ppm. No abdominal symptom was developed and increased intestinal gas was not found on the plain radiograph (Fig. 5D). Even when a diet order progress to solid foods, the patient did not complain of abdominal symptoms other than flatus and intestinal gas on the plain radiograph was not worsened (Fig. 5E). Although fasting breath H2 concentrations kept more than 10 ppm, the patient left hospital because of disappearance of abdominal symptoms.

## Discussion

H2 breath tests have been used to evaluate intestinal transit, bacterial overgrowth, and disaccharidase deficiency.^5^–^12^ It is based on the ability of the anaerobic microflora of the colon to ferment carbohydrate that has traveled unabsorbed through the small intestine, and to produce H2. This H2 is transported to the lungs and exhaled in the breath. A delay in intestinal transit time can maximize the intestinal residence time of carbohydrate and exposure time of the colonic bacteria to carbohydrate. Therefore, production of H2 gas in the intestinal tract may be continued for longer time by prolonging the contact of intraluminal contents with the intestinal bacteria. Although intestinal dysmotility is closely associated with H2 gas production, intraluminal gas production does not occur unless the intraluminal food residues reach the site where intestinal bacteria exists.

The patient who has both small bowel obstruction and small bowel bacterial overgrowth should have an increased H2 level in exhaled air, whereas a breath H2 level could not be increased if the patient with small bowel obstruction does not have small bowel bacterial overgrowth. In case 1, low levels of breath H2 before treatment might indicate that unabsorbed food residues did not reach the caecum due to small bowel obstruction. It was thought that improvement of intestinal passage after treatment resulted in the arrival of intraluminal contents at the caecum and H2 gas production by colonic bacteria. Actually, when small bowel gas decreased and a small amount of colonic gas was demonstrated on the plain abdominal radiograph on the fifth day after admission, the breath H2 concentration increased from 1 to 6 ppm. This change in breath H2 level may possibly reflect the movement of intraluminal contents from small intestine to the right colon. After that, this value reduced again to the baseline the next day and kept the low level (1–2 ppm) for a few days, suggesting that colonic fermentation has been finished because intraluminal contents have been consumed completely by the reaction. The breath H2 concentration increased markedly to 19 ppm one day after supplying a liquid meal despite the patient had no abdominal symptoms and intestinal gas was not increased on the plain abdominal radiograph. This suggests malabsorption of a liquid meal by which unabsorbed carbohydrates reach the colon and is utilized by fermentation, resulting in increased breath H2 levels. Although diets progressed from liquids to solids as tolerated, breath H2 concentrations were decreased to 2 ppm 5 days after supplying meals. Digestive and absorptive function is considered restored gradually. This is supported by the fact that the patient did not complain of any abdominal symptoms during a routine progressive diet and intestinal gas decreased continuously.

In case 2 who was treated with intravenous administration of 250 mg of erythromycin twice a day for the functional obstruction of small bowel, although the plain abdominal radiograph demonstrated a decrease of small-bowel gas, the breath H2 gas kept the low level. This is because the volume of small bowel contents is not enough to produce a great amount of H2 gas in the colon. Like case 1, on the next day after a clear-liquid meal was supplied, fasting breath H2 concentration increased rapidly to 22 ppm and gradually decreased to 9 ppm after 5 days despite the fact that the intestinal gas was unchanged on the plain radiographs. It is possible that digestion and absorption may be impaired and repaired shortly afterward. When diet progressed to rice porridge, fasting breath H2 concentration increased to 44 ppm, suggesting that a progressive diet might result in transient malabsorption. Generally, diets progressed gradually to a common solid food as tolerated in clinical practice. However, malabsorption linked to the progressive diets may be more common than expected.

It has been reported by several researchers that ingestion of a meal increases the volume of gas within the gut and this increment occurs relatively early after ingestion.^13^,^14^ Ingestion of the subsequent meal prompts ileal emptying and retained residues pass into the colon, giving rise to an early postprandial peak in breath H2 levels by colonic fermentation.^13^,^14^ Transient H2 increment found before serving meals in case 1 may reflect the passage of small bowel residues into the colon. In contrast to case 1, a rapid increase found in case 2 the day after serving could be resulted from unabsorbed food residues that passed into the colon and were fermented by colonic bacteria. Thus, the information on bowel movement or malabsorption of dietary food could be, to some extent, gotten noninvasively through changes in breath H2 concentration.

It has been controversial whether increased intestinal gas is linked to the abdominal symptoms. Koide et al.^15^ reported significant increased gas in a plain abdominal radiograph in patients with IBS. Although most of intestinal gas is produced by colonic fermentation in the right colon, it has been also unclear whether breath H2 gas, reflecting the intestinal gas produced by fermentation, is linked to the intestinal gas volume detected on plain radiographs. In both cases of the present study, breath H2 gas was not elevated while a markedly dilated small bowel was demonstrated on the plain abdominal radiograph. This suggests that increased intestinal gas is not related to on-going H2 production in the intestinal tract but also unabsorbed and stagnant intestinal gas produced previously. Morken et al.^16^ also showed that intestinal gas volume, as scored in plain abdominal radiographs, is not correlated with either abdominal symptoms or breath H2 excretion after lactulose challenge. In conclusion, a rapid increase of breath H2 concentration may reflect the movement of small-bowel contents to the colon in patients with small-bowel pseudo-obstruction or malabsorption following diet progression. Consecutive breath H2 analysis could give important information on the movement of intestinal food residues or the presence of malabsorption after meals. This noninvasive method may be another tool for evaluating the intestinal circumstances especially in patients with small bowel pseudo-obstruction and malabsorption during conservative treatment.
